# Latest evidence for the use of targeted temperature management in neurology

**DOI:** 10.1186/1471-227X-15-S1-A16

**Published:** 2015-06-24

**Authors:** Rainer Kollmar

**Affiliations:** 1 Klinik für Neurologie und Neurogeriatrie am Klinikum Darmstadt, Germany

## 

Acute brain injury induces a complicated cascade of deleterious pathways that can be counteracted by therapeutic hypothermia (TH) in the experimental setting [1]. While there is no level I indication for TH in neurology, TH is in principle useful for these indications if applied appropriately.

## Ischemic stroke

While fever is harmful in acute stroke, no clinical study showed improved outcome by antipyretic treatment – most likely because of poor antipyretic management [1]. In large ischemic stroke, post-ischemic brain edema and ICP are major therapeutic targets. A limited number of case series indicate feasibility and safety of TH (48 to 72 hours) in large ischemic stroke. However, rewarming led often to rebound of previously well controlled ICP and hernation [1]. Knowing the natural course of edema in large ischemic stroke, the duration of TH was chosen too short to maintain appropriate ICP control. After publication of a successful randomized controlled trial for hemicraniectomy in malignant stroke, TH might remain an experimental therapeutic option in large ischemic stroke which appears in more than the territory supplied by the middle cerebral artery. In this case, TH and hemicraniectomy could be combined and TH should last at least for more than 72 hours. Patients with acute ischemic stroke (>6 hours from onset) might in principle benefit from TH [1,2]. However, available data are limited and showed feasibility and safety of TH, but are underpowered for efficacy. Ongoing stroke trials include awake patients who are treated by an individual target temperature of 36 to 33°C [2]. Shivering and discomfort are counteracted by external warming and the use of pharmacological agents (opioids, buspirone, or/and dexmedetomidine). So far, both ongoing trials suffer from slow recruitment which indicates the complex procedure.

## Intracerebral hemorrhage

The outcome after large deep ICH is poor. Surgical and medical treatment do not improve outcome essentially. Beside the size of hematoma, the clinical condition is complicated by perihemorrhagic edema, which increases over more than 10 days [3]. Additionally, the size of perihemorrhagic edema is associated with the size of hematoma volume. Two case series showed that early TH (35°C for 10 days) was feasible and safe. TH was able to control ICP and prevented perihemorrhagic edema increase measured by repeated cranial CT. Moreover, neurological outcome and survival were superior compared with a historical control group [3]. At present, a German–Austrian controlled randomized clinical trial investigates efficacy in large ICH.

## Subarachnoid hemorrhage

Experimental data showed that TH was neuroprotective in SAH and reduced vasospasm, DCI and brain edema. Clinical data on the use of TH in SAH are limited. In a recent study without a control group, TH was used as a rescue therapy in patients with severe SAH and was feasible [4]. Smaller studies indicate that TH can reduce mean blood flow velocity in vessels with vasospasm and potentially improve outcome including less DCI.

In conclusion, TH is still a promising treatment option for acute brain injury. The use of TH has to be adapted to the specific targets of each condition and transferred to clinical studies.
